# Eye yoga for glaucoma: recovery of vascular dysregulation and visual field function—a randomized controlled trial

**DOI:** 10.1007/s13167-024-00389-x

**Published:** 2024-12-19

**Authors:** Wanshu Zhou, Luisa Fricke, Bernhard A. Sabel

**Affiliations:** Institute of Medical Psychology, Medical Faculty, Otto-von-Guericke University of Magdeburg, Leipziger Straße 44, 39120 Magdeburg, Germany

**Keywords:** Eye yoga, Glaucoma, Vision restoration, Flammer syndrome, Retinal vascular dysregulation, Ocular blood flow, Health risk assessment, Mental stress, Stress management, Psychosomatic diseases, Predictive and preventive personalized medicine (PPPM / 3PM), Neurovascular coupling, Primary open-angle glaucoma, Eye movement exercises, Meditation, Mindfulness, Quality of life

## Abstract

**Purpose:**

Because stress can aggravate vascular dysregulation (VD) in primary open-angle glaucoma (POAG), stress reduction by eye yoga (EY) was studied if this predictive, preventive, and personalized medical (3PM) approach could help normalize intraocular pressure (IOP), retinal vessel dynamics, and visual fields (VF).

**Patients and methods:**

POAG patients were randomized to an EY (*n* = 15) or control group (*n* = 12). EY was practiced daily for 1 h for 1 month at home using an iPod-audio guide while control patients read relaxing books daily.

**Results:**

After intervention, EY patients, but not controls, showed a 6.4% IOP reduction (*p* = 0.027) and had significant VF improvements (*p* < 0.001). After EY, pattern deviation recovered in VF regions where small microvessels showed reduced vasoconstriction (artery: *p* = 0.012; vein: *p* = 0.042) and improved mean artery diameter recovered significantly (*p* = 0.015). When pooling data of both groups, recovered VF regions, but not non-recovered fields, showed significantly larger arterial diameter gains (2.4 [− 0.3–5.3] MU) with no adverse events.

**Conclusions and 3PM recommendations.:**

Because EY reduces vasoconstriction and improves VF function in POAG, we propose the “eye ball retraction theory,” whereby ocular muscle tension is induced by mental stress which is a contributing mechanism, or even the key mechanism, of POAG. Reducing stress by relaxation is therefore a remedy for it improves blood flow as the fundamental mechanism of vision recovery and restoration. VD reduction is therefore a valuable therapeutic target for glaucoma care and eye yoga home exercises are a safe and effective complementary 3PM method of POAG care.

**Supplementary Information:**

The online version contains supplementary material available at 10.1007/s13167-024-00389-x.

## Introduction

Vision loss in glaucoma results from central nervous system damage of the retina, optic nerve, and brain, but the prognosis is pessimistic as the associated visual field (VF) loss is considered irreversible and progressive [1, 2]. Though vision can recover to some extent following IOP reduction [3–12], vision training [13–15], or microcurrent stimulation [16, 17], the mechanisms of recovery are still unclear.

According to the “residual vision activation theory” [18], vision recovery is explained by synaptic plasticity in the retina and reorganization of brain functional connectivity networks [18–24] which are thought to enhance visual/perceptual processing. But besides *neuronal* reorganization, we should also consider *vascular* mechanisms because their role in vision recovery is still unknown.

It is well known that the vascular state is a key component of glaucoma pathology and other eye and brain disorders [25, 26]. Especially normal tension glaucoma patients suffer from vascular dysregulation (VD) with typical signs or symptoms, collectively termed the “Flammer-Syndrome (FS)” [27–34]. The symptoms include cold hands or feet, prolonged sleep onset time, prolonged blood flow cessation in finger capillaries after cooling, autoregulation problems of ocular blood flow (OBF), increased retinal venous pressure with increased vessel stiffness, and altered activity of the autonomic nervous system (heart beat-to-beat variation [19]). It is expected that the resulting microvessel perfusion problems deprive energy-hungry neurons of oxygen and nutrients, disturbing neuronal activity and brain network synchronization which is tightly intertwined with blood flow by “neurovascular coupling” (NVC) [35–37]. This energy deprivation impairs neuronal signaling (propagation of action potentials), and visual processing in the retina and/or brain is disturbed or lost. We propose that besides neuronal death this “hypo-metabolic” state may be a primary “root cause” of neuronal dysfunction in primary open-angle glaucoma (POAG) which may either kill neurons in case of no energy supply or only “silence” neurons that are hypo-metabolic [38].

### Methods to implement 3PM: stress-related VD and new therapeutic target

Conventional glaucoma treatment focuses solely on controlling IOP through drugs or surgery. But many patients do not respond well to the treatment because vision loss progresses despite well-controlled IOP. In line with the principles of predictive, preventive, and personalized medicine (3PM) [39–42], consideration of other individual risk factors such as mental stress versus relaxation is vital for reducing or stopping progression and/or inducing restoration and repair.

Stress may be one major cause of vasoconstriction. Patients showing symptoms of the Flammer syndrome such as VD tend to have stress-prone personality dispositions with low-stress resilience, as they are particularly vulnerable to receiving bad news that their vision loss progresses which creates even more stress by anxiety and fear of going blind in a downward vicious cycle as follows:

$$\begin{array}{l}{\varvec{S}}{\varvec{t}}{\varvec{r}}{\varvec{e}}{\varvec{s}}{\varvec{s}}\boldsymbol{ }->{\varvec{e}}{\varvec{y}}{\varvec{e}}\;\boldsymbol{ }{\varvec{m}}{\varvec{u}}{\varvec{s}}{\varvec{c}}{\varvec{l}}{\varvec{e}}\;\boldsymbol{ }{\varvec{t}}{\varvec{e}}{\varvec{n}}{\varvec{s}}{\varvec{i}}{\varvec{o}}{\varvec{n}}/{\varvec{v}}{\varvec{a}}{\varvec{s}}{\varvec{o}}{\varvec{c}}{\varvec{o}}{\varvec{n}}{\varvec{s}}{\varvec{t}}{\varvec{r}}{\varvec{i}}{\varvec{c}}{\varvec{t}}{\varvec{i}}{\varvec{o}}{\varvec{n}}\boldsymbol{ }->{\varvec{s}}{\varvec{i}}{\varvec{l}}{\varvec{e}}{\varvec{n}}{\varvec{c}}{\varvec{i}}{\varvec{n}}{\varvec{g}}\;\boldsymbol{ }{\varvec{n}}{\varvec{e}}{\varvec{u}}{\varvec{r}}{\varvec{o}}{\varvec{n}}{\varvec{s}}\boldsymbol{ }->{\varvec{l}}{\varvec{o}}{\varvec{w}}\;\boldsymbol{ }{\varvec{v}}{\varvec{i}}{\varvec{s}}{\varvec{i}}{\varvec{o}}{\varvec{n}}\boldsymbol{ }->\\ \varvec{m}\varvec{o}\varvec{r}\varvec{e}\;\varvec{s}\varvec{t}\varvec{r}\varvec{e}\varvec{s}\varvec{s}->\dots ..->\varvec{m}\varvec{o}\varvec{r}\varvec{e}\;\varvec{v}\varvec{i}\varvec{s}\varvec{i}\varvec{o}\varvec{n}\;\varvec{l}\varvec{o}\varvec{s}\varvec{s}\dots \text{and so on}\end{array}$$

Personalized medicine approaches could integrate individual stress and VD conditions with other clinical data like IOP to tailor preventive or therapeutic interventions to optimize health outcomes on a personalized basis.

### Working hypothesis in the framework of 3PM

To evaluate if mental stress may be a root cause of POAG [38] in the framework of 3PM, we countered stress by relaxation to learn if this improves vascular health and VF function. Indeed, meditation is already known to reduce IOP, lower stress hormone levels, alter gene expression, and improve quality of life [43–45]. Even short-term ocular exercises and yoga-based lifestyle intervention lower IOP [46, 47]. If we gain a better understanding of how IOP, blood flow, and mental stress factors are related, we submit that it will help in advancing 3PM approaches by predicting and preventing disease progression and developing more personalized treatment plans. As we now show, 1 month of stress-reducing relaxation with daily eye yoga exercise (EY) (the *independent* variable) can improve vascular health and restore VF function (the *dependent* variable).

## Materials and methods

### Experimental design

**Fig. 1:**
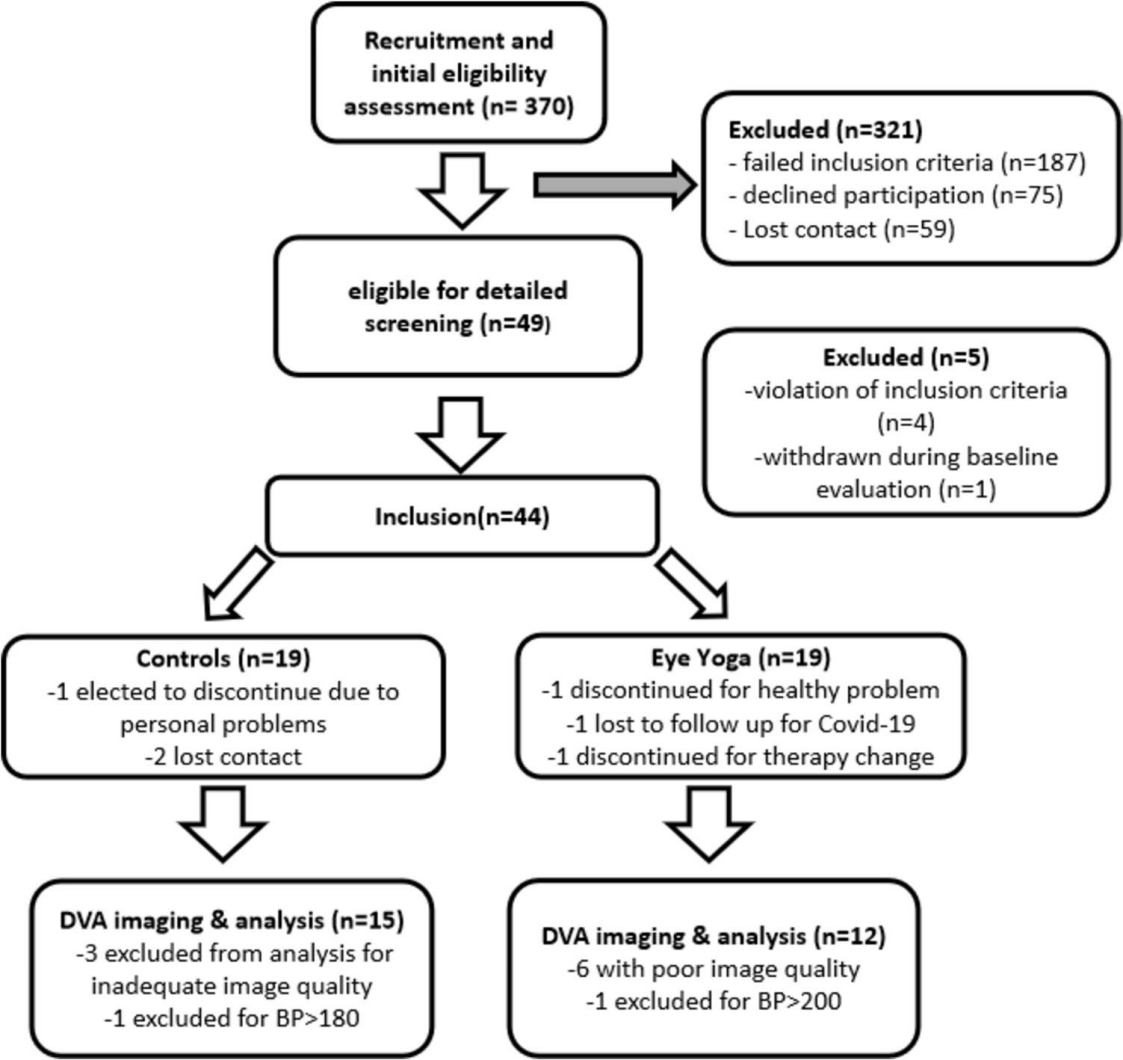
Flow diagram of patient selection and DVA analysis. Of 49 eligible patients, 4 did not meet all inclusion criteria (1 no visual field defect, 2 angle closure glaucoma, and 1 unable to read), and 1 withdrew during baseline evaluation. During the treatment phase, 3 subjects discontinued for personal problems, therapy change, or health problem; 1 subject lost for COVID-19; and 2 lost contact. In the analysis stage, 9 had poor DVA image quality at baseline or post-treatment (8 poor refractive pathway, and 1 had sick feeling), and 2 excluded for temporarily high BP

To participate in our prospective, randomized, double-blind, placebo-controlled clinical trial (flow diagram and study design are shown in Fig. 1), participants gave written informed consent and were then 1:1 randomized by lot to either the EY or “active” placebo group by the unblinded project manager (L.F.). The local ethics committee approved the study plan in compliance with the Declaration of Helsinki (registered at ClinicalTrials.gov: NCT04037384). Patient recruitment and follow-up were carried out from 8/2019 to 12/2022 with diagnostic evaluations at BASELINE and after the 30-day treatment (POST) by study nurses blinded regarding group identities in Institute of Medical Psychology, Faculty of Medicine, University of Magdeburg, Germany. Each diagnostic evaluation took 2 days and POST evaluation occurred within 1 week after treatment.

Inclusion criteria: residual vision, ability to read, and POAG known for at least 6 months. Exclusion criteria: cataract, narrow-angle glaucoma, acute autoimmune disease, neurological or mental disease (e.g., epilepsy, schizophrenia, and depression), addiction, hypertension > 160/100 mmHg, other ocular diseases affecting nerve tissue, pathological nystagmus, unoperated tumors or tumor recurrence, known history of abnormalities in EEG, or signs of photosensitivity, and pregnancy. To avoid the confounding variable of the IOP-lowering medications, all patients were asked to continue their prescribed drug use during the study period. While some prostaglandin analogs may minimally increase ocular blood flow, patient randomization was used to avoid a systematic confound.

### Patient sample description

Because there have been no detailed reports on VF after EY, we used short-term IOP change after yoga ocular exercise to estimate sample size (IOP decreased from 16.25 ± 2.48 mmHg to 14.50 ± 2.58 mmHg) [47]. The required sample size was 20 per group (95% confidence level and 90% power). Assuming a 10% drop-out rate, we recruited 44 patients (22 per group). Patients were asked to continue their prescribed drugs (no “wash-out”).

### Outcome measures

Retinal vessel regulation and VF function analyses reported here were *secondary* outcome measures. The primary outcome criterion (results to be reported elsewhere) was microsaccade dysfunction improvement.

### Eye yoga intervention

EY is known for thousands of years in Indian Ayurveda traditional medicine, and Chinese “ocular gymnastics” or Qi Gong are still popular today and compulsory for schoolchildren in some Asian cultures. Our patients learned EY before baseline evaluation and were asked to practice daily for 30 days at home (15 min morning and evening) using an iPod-based audio app that systematically guided them (i) to perform systematic eye movements to stretch their ocular muscles in all directions, (ii) periorbital and facial massage, (iii) iris relaxation in darkness (“palming”), and (iv) controlled breathing meditation (“Pranayam”) [48]. Meditation is a well-accepted mind relaxation technique that normalizes stress hormone levels and inflammation markers [51]. Controls were asked to daily read relaxing/entertaining books for 30 min (“active placebo”).

### Visual field and IOP examinations

VF testing pre- (baseline) and post-intervention was done with Twinfield-2 Perimetry (Oculus/Germany) using 8-pattern setting (0–30°/66 points; fast threshold strategy; size III/white stimuli). It quantifies age-adjusted global (mean deviation, MD) and local VF defects at each test position (pattern deviation, PD). IOP was measured pre/post-intervention (averaged triplicates) with iCare HOME Tonometer (Icare, Vantaa, Finland). To reduce test–retest variability, all VF and IOP measurements were made in the morning, in the same location, and in the same sequence. All subjects had a POAG history and experience with repeated IOP and VF measurement which limits any learning effect confounds.

### Dynamic vessel analysis

**Fig. 2:**
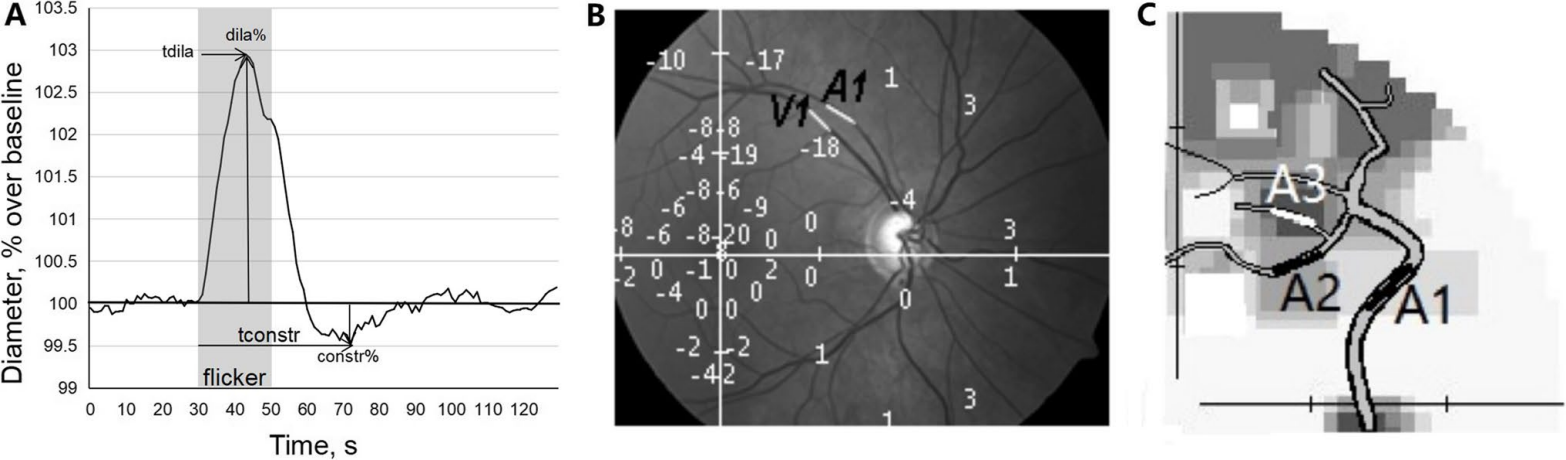
**A** Parameters of retinal vascular response to flickering light stimulation: “maximal dilation” (dila%): peak dilatation during 20 s flickering period compared to PFB; “time to maximal dilation”(tdila): time to peak dilatation after flickering onset; “maximal constriction” (constr%): peak constriction after flicker onset compared to PFB; “time to maximal constriction” (tconstr): time to reach peak constriction. **B** and **C** Compound analysis of retinal vessel tree, VF function (in dB), determination of branch order for arteries (A1–3) and veins (V1–3). **B** is an example of a retinal image with a superimposed 8 (0–30°/66 points) VF test pattern (retina image resolution: 2452 × 2056 pixels; VF size: 50° × 42°). The linear scaling was 2,452/50 = 49 pixel/degree. The 8 (0–30°/66 points) VF pattern (same linear scaling) was centered on the macula, and vessel segments were assigned to a nearest PD (vertically flipped to match orientation). This example shows two vessel segments (A1, V1) that were assigned to their nearest PD value (− 18 dB). **C** Illustration of branch order analysis showing how branch levels 1–3 were matched with the VF defect values: A1 (branch order 1; (BO-1)) was located in the mild defect, A2 (BO-2) in a moderate, and A3 (BO-3) in a severely damaged VF sector

Retinal vessels were imaged [49, 50] in a darkened room with a dynamic vessel analyzer (DVA) (Imedos, Jena, Germany) with blood pressure (BP) before and after DVA, and IOP tests prior and after pupil dilation, and after completing DVA. Following the first pupil dilation (Mydriatics 1 ml solution containing 5.0 mg tropicamide, Pharma Stulln GmbH, Germany), the fundus of the central 30° of visual angle was video-recorded with the DVA in each eye comprising three phases: (i) a 30-s pre-flicker baseline (PFB), (ii) three 20-s stimulation periods of diffuse luminance 12.5 Hz light flickers, and (iii) three 80-s post-flicker periods. Using DVA software, vessels were analyzed regarding absolute vessel diameters (measuring unit: MU = micrometers in Gullstrand’s eye) and dilation dynamics. To establish PFB diameter (100%), the pre-flicker 50-s recordings (including 20 s from the last post-flicker periods) were pooled and averaged. Based on the diameter response to flickering light, expressed in percentage change over PFB, different parameters were calculated (see Fig. 2A for details). DVA measurement was also conducted in the morning to reduce test–retest variability.

Vessel dynamics was measured in multiple retinal locations to differentiate branch hierarchies (smaller vs. larger vessels) and different VF areas of the intact and damaged eye for areas of mild, moderate, or severe defect depth. Vessel segments were only analyzed if (i) the image had sufficient contrast for artefact free recording; (ii) no crossing, bend, or vessel bifurcation; (iii) located outside a circular area of one disc diameter from the optic disc center; and (iv) > 1 vessel diameter distance to neighboring vessels.

### Retinal vessel topography

To match VFs with vessels, retina photographs were obtained by fundus camera (50°, Zeiss, Jena, Germany) digitized at 2452 × 2056 pixel resolution in a linear scaling of 49 pixels/degree (reference: emmetropic eye). This image was superimposed on the 8-pattern VF plots of identical scaling (49 pixels/degree) using Photoshop so the VF center could be aligned to the fovea. After vertical flip of the VF chart, the retina fundus image could be matched with the specific PD value (VF depth) and the nearest PD value was selected (Fig. 2B). Only vessel branch segments were chosen where both baseline and post-treatment PD values were available. We next combined the data of the EY and reading group to calculate the average parameters of three PD sub-groups according to their baseline perimeter results (mild defect < − 6 dB, moderate defect − 6 to − 12 dB and severe defect ≤ − 12 dB).

### Retinal vessel branch level analysis

All vessel segments were classified by branch order (BO): BO-1 are the largest vessels emerging from the papilla; BO-2 those branching off first from BO-1, and BO-3 all those beyond the next bifurcation, i.e., higher-up, smaller branches (Fig. 2C). Of note, many BO-3 branches were too small (faint) for DVA-detection. To determine how vessel morphology and dynamics relate to VF recovery, we also pooled the data of both study groups and calculated the absolute difference (Δ, pre–post) of all vessel parameters as a function of PD recovery defined as improvement > 3 dB, i.e., irrespective of intervention (Table 2).

### Statistical analysis

Data were analyzed with SPSS 26 (IBM, New York, USA) and Matlab (MathWorks, Natick, USA). Chi-squares test was applied for gender analysis, independent-samples *T*-test for two-group comparisons, and paired-samples *T*-test for pre- and post-treatment comparison for normally distributed samples. Otherwise, we used Mann–Whitney *U*-test for two-group comparisons and Wilcoxon test for pre- and post-treatment comparison. Because our study was of exploratory nature, alpha was not adjusted for multiple comparisons to avoid type-1 errors, and vessels were treated as independent samples. MD, IOP, and vessel diameter were tested hypothesis-wise (one-sided *p*-value).

## Results

### Demographic parameters, blood pressure, IOP, and visual field function

**Fig. 3:**
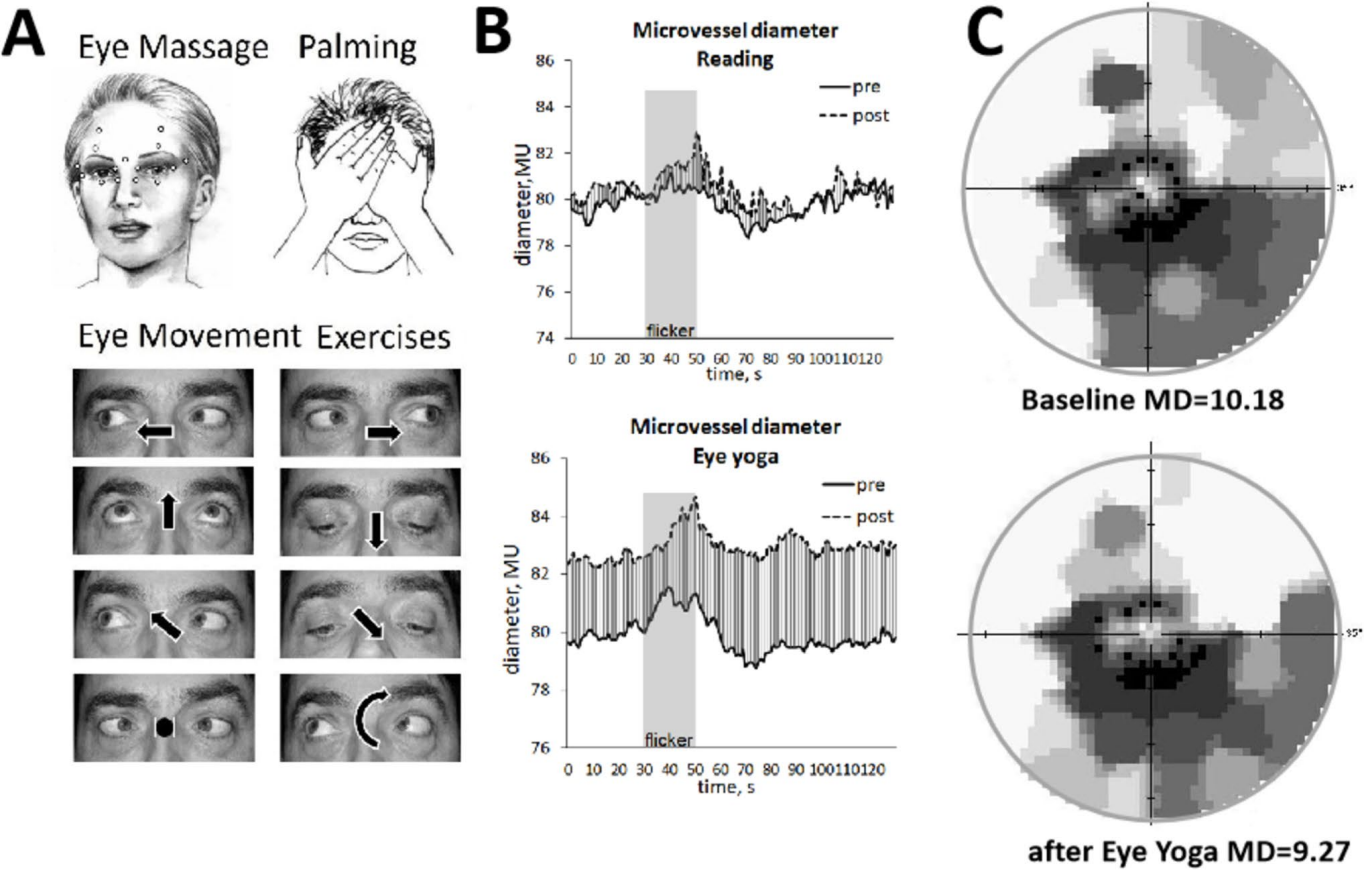
**A** Elements of the eye yoga exercises (meditation not shown). **B** Averageof BO-3 artery diameter and its response to flicker light in the eye yoga or reading (control) group. **C** Example of VF recovery in all four quadrants of an excellent eye yoga responder; MD means mean deviation

Nineteen patients each in the EY and reading group completed baseline evaluation, but some either did not complete the trial, and in others, the DVA analysis was not possible (see Fig. 1). Finally, 15 EY and 12 control patients had complete data sets with comparable mean age, gender ratios, and BP of both groups (Table 1). Both patient groups were comparable regarding glaucoma stages (MD: EY 4.7 dB, Reading 3.3 dB, *p* = 0.201) and concomitant cardiovascular health because those with significant cardiovascular diseases were excluded from study participation. Both group’s baseline parameters were similar to those of the lost subjects, except that IOP of lost patients (16.3 [13.8,18.9] mmHg) was significantly higher than those retained (13.6 [12.4,14.7] mmHg), though both were still in the normal range. Hypothesis-wise testing revealed a significant IOP reduction and improved VF function (MD) only in the EY group but not in the control group (pre/post 12.8 [10.8, 14.9]/12.8 [10.7, 14.9] mmHg, *p* = 0.465). We found a 6.4% IOP reduction after EY (pre 14.1 [12.7, 15.5]; post 13.2 [11.8, 14.7] mmHg; *p* = 0.027). Regarding VF function, controls showed a non-significant 4% MD improvement (pre/post 4.7 [2.7, 6.7]/4.5 [2.3, 6.7] dB, *p* = 0.217) but EY a significant 24% sensitivity improvement from 3.3 dB [1.3, 5.4] to 2.5 dB [0.4, 4.6] (*p* < 0.001) (Fig. 3C) (Table 1).

**Table 1:** BP, IOP, MD, and BO-3 vessel parameters (significant values display in underlined italic)

		Normal^a^	Reading	Eye yoga	*p*^b^
Nnumber of subjects		22	12	15	
Number of eyes		43	21	29	
Age (SD), year		66.4 (8.8)	67.1 (8.3)	62.8 (12.7)	0.331
Gender(female:male)		14:8	4:8	9:6	0.168
Systolic pressure (SD), mmHg	Pre	132.1 (12.7)	130.8 (14.9)	131.7 (14.5)	0.864
	Post	-	128.9 (17.4)	132.9 (14.3)	0.516
	*p*^c^	-	0.618	0.654	
Diastolic pressure (SD), mmHg	Pre	82.6 (8.7)	76.3 (12.7)	80.7 (5.7)	0.284
	Post	-	73.3 (11.4)	80.5 (6.1)	0.067
	*p*^c^	-	0.135	0.872	
IOP (SD), mmHg	Pre	14.3 (3.7)	12.8 (4.6)	14.1 (3.7)	0.281
	Post	-	12.8 (4.6)	13.2 (3.8)	0.733
	*p*^c^	-	0.465	*0.027*	
Mean deviation (SD)^d^, dB	Pre	-	4.7 (4.4)	3.3(5.4)	0.201
	Post	-	4.2 (5.1)	2.5(5.4)	0.178
	*p*^c^	-	0.217	< *0.001*	
BO-3 artery					
Vessel number		87	23	29	
Pattern deviation (SD), dB	Pre	-	− 1 (2)	− 2.7 (2.9)	*0.033*
	Post	-	− 0.8 (2.6)	− 1.2 (4)	0.296
	*p*^c^	-	0.386	*0.012*	
Diameter (SD), MU	Pre	83.7 (12.4)	79.7 (13.9)	79.9 (10.5)	0.530
	Post	-	80.4 (14.1)	82.5 (12.1)	0.596
	*p*^c^	-	0.273	*0.015*	
dila% (SD), % over baseline	Pre	3.6 (2.4)	3.2 (2.4)	4.5 (4.2)	0.473
	Post	-	3.9 (5.2)	4 (3.1)	0.106
	*p*^c^	-	0.808	0.820	
constr% (SD), % over baseline	Pre	− 3.0 (2.0)	− 3.4 (2)	− 3.2 (2.2)	0.473
	Post	-	− 3.6 (3.6)	− 3.2 (1.8)	0.615
	*p*^c^	-	0.26	0.854	
tdila (SD), s	Pre	13.3 (5.3)	39 (5.6)	42.9 (5.6)	*0.032*
	Post	-	42.4 (5.7)	42.1 (6.9)	0.950
	*p*^c^	-	*0.011*	0.809	
tconstr (SD), s	Pre	46.4 (26.0)	88.2 (30)	84.5 (26.8)	0.451
	Post	-	84 (30.7)	81.4 (28.3)	0.733
	*p*^c^	-	0.7	0.746	
BO-3 vein					
Vessel number		71	23	21	
Pattern deviation (SD), dB	Pre	-	− 1.9 (3.3)	− 2 (2.7)	0.961
	Post	-	− 2 (6.3)	− 0.7 (3.8)	0.902
	*p*^c^	-	0.188	0.042	
Diameter (SD), MU	Pre	88.1 (20.7)	84.8 (14.1)	83.7 (12.1)	0.743
	Post	-	82.5 (14.1)	84 (11)	0.568
	*p*^c^	-	0.065	0.379	
dila% (SD), % over baseline	Pre	3.7 (2.0)	3.6 (2.7)	5.7 (5.9)	0.215
	Post	-	2.4 (1.4)	5.4 (4.4)	*0.027*
	*p*^c^	-	0.181	0.848	
constr% (SD), % over baseline	Pre	− 2.4 (2.3)	− 2.8 (1.8)	− 2.8 (1.4)	0.528
	Post	-	− 2.2 (1.6)	− 3 (1.8)	0.089
	*p*^c^	-	0.23	0.694	
tdila (SD), s	Pre	15.1 (5.2)	41.3 (6.4)	46.5 (4.2)	*0.002*
	Post	-	43.4 (5.3)	41.8 (7.1)	0.442
	*p*^c^	-	0.337	*0.007*	
tconstr (SD), s	Pre	47.1 (35.2)	93 (29.9)	89.4 (32)	0.894
	Post	-	86.8 (33.1)	83.8 (36.9)	0.697
	*p*^c^	-	0.315	0.639	

^a^Quoted from our last publication [38]. ^b^Chi-squares test for gender analysis, Mann–Whitney *U*-test or independent-samples *T* test for other comparisons between reading and eye yoga groups. ^c^Wilcoxon test or paired *T* test for pre- and post-treatment comparison. ^d^Absolute value. Vessel results reported here are only from the smallest measurable microvessels of branch order level 3 (BO-3). *MU*, measuring unit. 1 MU is approx. 1 µm

### Vascular morphology and dynamics

We already reported our baseline data showing vessel diameter shrinkage in glaucoma patients compared to healthy controls [38]. But unexpectedly, there was no significant difference between those healthy controls and our EY patients (suppl.Tab.1). This could possibly be explained by a sampling bias caused by the limited DVA-optics resolution or loss of small (BO-2/BO-3) microvessels in the patients.

BO-3 microvessels were of special interest because they are the smallest and still visible microvessels, and they have the largest surface:volume ratios. This makes them particularly vulnerable to vasoconstriction with the greatest (4th-power) impact on OBF. Hence, subtle microvessel diameter changes (decline or recovery) have the greatest impact on local OBF and neuronal function. Indeed, our analysis revealed no consistent signs of recovery of vessel morphology or dynamics in larger BO-1/BO-2-branches. But post-intervention, the smallest BO-3 EY vessels had significantly increased arterial diameters and faster venous dilation, whereas placebo patients had slower arterial dilation (Table 1).

When matching visual dysfunction (PD) with vascular status, EY patients had significantly improved visual sensitivity in locations where signs of arterial and venous (BO-3) microvessels diameter recovery were observed. The respective visual sensitivity improvement for retinal regions showing recovered arteries was − 2.7 [1.6, 3.8] to − 1.2 [0.3,2.8] dB (*p* = 0.012) and for veins − 2.0 [0.8,3.2] to − 0.7 [− 1.0, 2.5] dB, *p* = 0.042). While arterial diameter significantly increased after EY from 79.9 [75.9, 83.9] to 82.5 [77.9, 87.1] MU (*p* = 0.015), the reading group showed no such changes (Table 1, Fig. 3A). Furthermore, after intervention, time-to-peak dilation was significantly slower in arteries of controls and significantly faster in veins after EY (Table 1).

### Vascular state and visual field defect depth

We also studied if vessel state or dynamics vary as a function of VF dysfunction (defect depth) at baseline. When data of both groups was combined, there was no apparent recovery in regions of mild impairment (ceiling effect?), but significant VF recovery was observed in regions of moderate and severe of damage (see Suppl.-Tab. 2). Unexpectedly, the vessel parameters were comparable across different VF severity levels.

**Table 2:** Vessel parameters in recovered vs. non-recovered visual field sectors (Δ = post–pre; significant values display in underlined italic)

		Recovered	Non-recovered	p*
Artery				
Vessel number	Reading	16	108	
	Yoga	23	152	
	Pooled	39	260	
ΔPattern deviation (SD), dB	Reading	6.2 (2.6)	0 (2.2)	< *0.001*
	Yoga	7.7 (3.6)	− 1.5 (4)	< *0.001*
	Pooled	7.1 (3.3)	− 0.9 (3.5)	< *0.001*
ΔDiameter (SD), MU	Reading	− 2.2 (4)	− 1 (5.2)	0.188
	Yoga	2.4 (6.5)	− 0.1 (4.9)	*0.014*
	Pooled	0.5 (6)	− 0.5 (5.1)	0.142
Δdila% (SD), % over baseline	Reading	− 1.2 (3.3)	0.1 (3.3)	0.365
	Yoga	0.3 (3.5)	− 0.3 (3)	0.236
	Pooled	− 0.3 (3.5)	− 0.2 (3.1)	0.685
Δconstr% (SD), % over baseline	Reading	0 (1.6)	0.1 (2.8)	0.964
	Yoga	− 0.6 (2.1)	0.5 (2.2)	0.092
	Pooled	− 0.4 (1.9)	0.3 (2.5)	0.179
Δtdila (SD), s	Reading	− 0.3 (5.4)	1.5 (7.6)	0.282
	Yoga	− 0.2 (7.9)	0.3 (7.9)	0.791
	Pooled	− 0.2 (6.9)	0.8 (7.8)	0.45
Δtconstr (SD), s	Reading	0.4 (38.1)	− 0.6 (40.1)	0.928
	Yoga	− 2.8 (40.6)	− 3.8 (35.1)	0.901
	Pooled	− 1.5 (39.1)	− 2.5 (37.2)	0.571
Vein				
Vessel number	Reading	16	105	
	Yoga	30	139	
	Pooled	46	244	
Pattern deviation (SD), dB	Reading	7.8 (3.3)	0.1 (2.7)	< *0.001*
	Yoga	6.8 (2.9)	− 1.2 (3.5)	< *0.001*
	Pooled	7.1 (3.1)	− 0.6 (3.3)	< *0.001*
Diameter (SD), MU	Reading	− 2.9 (3.5)	− 1.1 (7.1)	0.053
	Yoga	1 (5.4)	− 1.2 (5.2)	0.076
	Pooled	− 0.4 (5.2)	− 1.1 (6.1)	0.432
dila% (SD), % over baseline	Reading	0.1 (2)	0.2 (2.8)	0.603
	Yoga	− 0.3 (2.7)	0 (3.7)	0.57
	Pooled	− 0.2 (2.5)	0.1 (3.3)	0.345
constr% (SD), % over baseline	Reading	0.2 (1.6)	0.1 (1.9)	0.973
	Yoga	− 0.8 (2.6)	0.1 (2.5)	0.113
	Pooled	− 0.5 (2.3)	0.1 (2.2)	0.198
tdila (SD), s	Reading	0.3 (5.7)	0.7 (6.8)	0.794
	Yoga	− 2.7 (6.5)	− 1.2 (6.4)	0.497
	Pooled	− 1.7 (6.4)	− 0.4 (6.6)	0.616
tconstr (SD), s	Reading	− 5.5 (47.3)	− 3.5 (46)	0.777
	Yoga	− 1.4 (44.8)	− 5 (44.3)	0.939
	Pooled	− 2.8 (45.2)	− 4.3 (45)	0.902

Δ = post–pre. Recovered vessel are those located in VF sector where Δ pattern deviation > 3 dB. Non-recovered vessel ≤ 3 dB. *Mann–Whitney *U*-test or independent-samples *T* test for comparisons between reading and eye yoga group, and all patients, irrespective of treatment (pooled)

### Comparing vessels in recovered vs. non-recovered visual field sectors

To gain insight into vascular mechanisms of vision restoration/recovery, we explored how vascular changes relate to VF recovery of > 3 dB gain when the data of all patients were pooled, i.e., irrespective of invention (Table 2). In retinal areas with arterial recovery, the respective PD recovery was 6.2 [4.8, 7.6] dB in controls, 7.7 [6.1, 9.2] dB in EY, and 7.1 [6.0, 8.1] dB in both groups combined. No such change was noted in regions without arterial recovery (PD values 0.0 [− 0.4,0.4], − 1.5 [− 2.1, − 0.9], and − 0.9 [− 1.3, − 0.5] dB, respectively). VF regions of recovered veins showed similar results (*p* < 0.001). Recovered VF sectors in EY patients, but not controls, had increased *arterial* vessel diameters (2.4 [− 0.3, 5.3] MU gain) but no change in non-recovered VFs (− 0.1 [− 0.9, 0.7] MU) (*p* = 0.014). *Venous* vessel diameters in controls showed a trend of smaller diameters (*p* = 0.053) in recovered fields, whereas EY veins had a trend of enlarged diameters of 1.0 [− 1.1, 3.0] MU (*p* = 0.076, Fig. 3B).

### Adverse events

Both treatments were well tolerated and without any treatment-associated adverse events.

## Discussion

Though there are only three *PubMed*-listed EY studies in glaucoma, there is growing interest in relaxation techniques to lower IOP [51]. Following our earlier demonstration that vessel diameters are reduced in POAG patients which compromises OBF, our study is the first controlled trial showing that EY is safe and effective to lower IOP, normalize VD in microvessels, and improve VF function. Though a 3% diameter recovery seems small, according to Poiseuille’s law, vessel diameter has a 4th-power influence on blood flow. Therefore, an EY-induced 2.5 MU increase in recovered vessels translates to a 12.6% OBF restoration [(100% + 3%)^4^–100% = 12.6%], i.e., an increase reaching almost the 15% that POAG patient lost compared to healthy controls [38]. We hypothesize that this normalization of vasoconstriction has a (secondary) down-stream effect of reactivating hypo-metabolic (“silent”) neurons and provides the biological mechanism of vision restoration.

Interestingly, even squeezing the eyelids for a few minutes is enough to increase IOP [52], while ocular massage lowered mean IOP immediately by − 6.2 ± 1.9 mmHg, but this effect persisted only 5 min after the ocular massage with a mean reduction of − 3.8 ± 2.0 mmHg [53]. In contrast to the brief changes in IOP associated with eyelid squeezing or ocular-facial massage, our eye yoga involved slow, deliberate eye movements, ocular-facial massage, and deep breathing meditation to promote relaxation and reducing stress on a daily basis over the course of 1 month. We hypothesize that slow and deliberate eye movement contracting and stretch oculomotor and ciliary muscles reduces muscle tension (like body postures of yoga asanas). This then also relaxes retinal vessels and increases outflow of aqueous humor, thus lowering the IOP. Meditation, on the other hand, is a more general method to lower mental stress which activates the parasympathetic nervous system which alone can already lower IOP in glaucoma patients [45].

From a basic science perspective, our results confirm the hypothesis that stress should be considered a key *cause*, not only an *effect*, of glaucoma and that relaxation is a complementary technique to improve vision. This causality is justified because the independent variable (treatment using relaxation) altered the dependent variables IOP, vessel diameter, and VF function. Therefore, the widespread opinion that stress is a “non-issue” in glaucoma care needs revision for two reasons: first, glaucoma patients with VD tend to have personality dispositions of low-stress resilience, being worrisome, perfectionistic, ambitious, compulsive, and/or neglecting their own needs [54–56]. This, in turn, creates sudden or long-term mental stress triggering a psychosomatic response [57]. Second, the diagnosis of progressing vision loss triggers a pessimistic life perspective because the continuous anxiety and fear of going blind possibly accelerate progression (“dark thoughts—cloudy vision”), a downward spiraling (vicious) cycle [58, 59].

That stress negatively impacts all bodily organs is the basis of psychosomatic medicine, and, as we confirm, the eyes are no exception: first, stress increases stress hormones such as glucocorticoids [60], pro-inflammatory cytokines [61], and endothelin-1 [30, 62–64], and it reduced nitric oxide (NO) [65, 66], a vasodilatory neuropeptide lining rich in peri-vascular neurons and in the endothelium. Second, stress triggers sympathetic activation, increasing muscle tone (tension/constriction) not only of the perivascular muscles but possibly also of the oculomotor muscles. We submit that chronic vasoconstriction in POAG therefore continuously deprives retinal and brain neurons of oxygen and nutrients, lowering neuronal energy states and reducing visual processing [38, 58].

The most effective, long-known remedy for stress is relaxation. It is not only used for thousands of years in traditional medicine, but many relaxation programs are standard care in psychosomatic medicine, psychotherapy, psychiatry, and neuro-rehabilitation. But so far they are not used in standard care of low vision rehabilitation. Our study results confirm the benefits of relaxation techniques (here: eye exercises and meditation), and it provides the biological explanation why vision can be improved or restored: it improves vascular regulation and blood flow. This is in line with others showing that yogic ocular exercises lower IOP in the short- [47] and long-term [46] as does breathing (mindfulness) meditation which reduces stress- and inflammation biomarkers (cortisol, IL6, TNF-α, and ROS), it elevates β-endorphins, BDNF, and TAC and even alters gene expression [44, 45].

Based on our observations, we propose the following two-pronged theory of glaucoma which we term the “eye ball retraction theory”: first, increased (sympathetic) muscle tension shortens oculomotor muscles, pulling the eyes slightly backward against the eye socket of the skull which, in turn, increases IOP and constricts OBF in the optic nerve head (paling) and choroid, especially when intracranial and optic nerve subarachnoid space pressure is low [67, 68]. Second, stress may increase retinal perivascular smooth muscle tension, adding to the eye pressure-induced vasoconstriction.

This “eyeball-retraction-theory” might explain why relaxation by EY is a remedy for glaucoma: stretching the oculomotor muscles during eye movement exercises lowers their tension which has the advantage of lifting the pressure against the choroid, possibly reducing IOP [44, 45], normalizing the eye globe’s shape, and/or enhancing aqueous outflow. Relaxation of the perivascular smooth muscles on the other hand increases vessel diameter, normalizing OBF. This leads us to the proposition that the mechanism of vision restoration after eye yoga is primarily due to vascular recovery. If true, neuronal recovery (neuroplasticity) would only be an effect secondary to improved blood flow.

Though we recognize that the lumen regulation of venous vessels is different from that of arteries [69], the dilation of venous vessels observed in our study can be explained by eye yoga relaxation which may have reduced the perivascular smooth muscle tension. Venous dilation, unlike arterial regulation, is more passive but can still be influenced by changes in local blood flow and pressure dynamics [30]. Thus, relaxation-induced venous dilation could be a contributing factor to the overall improvements in ocular blood flow and visual function, though further studies are needed to clarify this issue.

Overall, although further study is needed to confirm the proposed mechanism and long-term effect of EY, our study offers potential avenues for personalized POAG therapy within the framework of 3PM: in contrast to an IOP-centric focus to explain glaucoma, we agree with the proposal of Josef Flammer [27, 29] that VD or vascular dysfunction is a key mechanism of open angle glaucoma which, according to our own suggestion, is compatible with the view that mental stress may be the underlying cause. We propose that in the future, glaucoma treatment should be tailored to the patients’ individual characteristics (blood flow, mental stress, whole body status, lifestyle, environmental factors, etc.) rather than focusing on the one and only risk factor, IOP. It would open the door for a more personalized, holistic approach to preventing progression of vision loss and optimization of restoration and recovery in low vision rehabilitation.

### Limitations and recommendations

Though our study sample is small, yet significant benefits of EY were observed which argues for a strong effect. Furthermore, some of our “active control” patients showed some vision recovery as well, possibly due to either normal variation, placebo effects, or the (non-specific) benefit of daily “time-out” relaxation. Whatever the mechanism, relaxation by EY is “complementary” (not an alternative) to standard IOP management. Future studies could test if EY can increase efficacy of drug therapies and/or improve a more optimistic mental state of mind.

### Toward prevention of glaucoma

Stress-related VD is a risk factor not sufficiently considered in clinical practice. The discovery of vascular recovery in this study once again reaffirms the role of the blood flow theory in the prevention of glaucoma. Future studies should therefore also consider the use of EY for preventing the occurrence and progression of glaucoma.

### Toward personalization of glaucoma treatment

In this study, we validated that EY, a psychosomatic therapy, can effectively reduce IOP, increase blood flow, and improve vision. Because of our results, we propose that EY is an effective complementary therapy for glaucoma. It offers the advantages of being harmless, non-invasive, side-effect free, and low-cost, enriching our means of treating glaucoma and contributing to personalized treatment of glaucoma.

### Toward predictiveness of glaucoma

In addition to these considerations, blood flow factors are one of the mechanisms underlying glaucoma. As we demonstrated, it is the improvement of stress-related vascular dysfunction which may be a key factor contributing to VF recovery in glaucoma. Future research should develop and establish models to predict the onset, prognosis, and possible recovery of glaucoma based on patients personalized assessment of blood flow function. In this manner, our findings may contribute to a paradigm shift from medicine which is just reactive to increase IOP or VF dysfunction in glaucoma by considering a more personalized (3PM) approach which goes beyond the traditional state of the art.

## Conclusion

Stress-reducing relaxation by eye yoga (combined eye exercises and meditation) can normalize vasoconstriction and improve VF in POAG. Though a study with larger sample size is desirable, we recommend that EY (including meditation) can already be adopted in low vision rehabilitation. It is safe and effective to improve VF dysfunction, possibly slowing down progression. And it may be a useful solution for patients who fail to respond to standard eye care comprising a new 3PM management of patients who suffer vision loss because of glaucoma. It add a new complementary dimension to standard eye care.

## Supplementary Information

Below is the link to the electronic supplementary material.

Supplementary file1 (DOCX 19 KB)

Supplementary file2 (DOCX 18 KB)

## Data Availability

Datasets can be made available to qualified requests.

## Abbreviations

POAG: Primary open-angle glaucoma
IOP: Intraocular pressure
EY: Eye yoga
VF: Visual field
VD: Vascular dysregulation
FS: Flammer syndrome
OBF: Ocular blood flow
NVC: Neurovascular coupling
EEG: Electroencephalogram
MD: Mean deviation
PD: Pattern deviation
DVA: Dynamic vessel analyzer
BP: Blood pressure
PFB: Pre-flicker baseline
MU: Measuring unit
BO: Branch order
NO: Nitric oxide
