# Supplementary material for: Eye yoga for glaucoma: recovery of vascular dysregulation and visual field function—a randomized controlled trial
Source: EPMA J. 2024 Dec 19;16(1):37–49. doi: 10.1007/s13167-024-00389-x (PMC11842685; doi:10.1007/s13167-024-00389-x)
Supplement: Supplementary file 1 — Supplementary file1 (DOCX 19 KB) [file 13167_2024_389_MOESM1_ESM.docx]

| **Supp. Table 1. Vessel parameters per vessel**  **(all vessel branch orders)** | | | | | |
| --- | --- | --- | --- | --- | --- |
|  |  | Healthy subjects*^a^* | POAG  reading | POAG  eye yoga | p^b^ |
| Artery |  |  |  |  |  |
| vessel number |  | 383 | 120 | 173 |  |
| Diameter(SD), MU | pre | 94.8(18.0) | 89.7(15.2) | 94.5(14.7) | 0.005 |
|  | post | - | 88.6(14.9) | 94.8(15.1) | <0.001 |
|  | p^c^ | - | 0.01 | 0.361 |  |
| dila%(SD), % over baseline | pre | 3.5(5.7) | 3.6(2.7) | 4.1(2.9) | 0.186 |
|  | post | - | 3.5(3.2) | 3.9(2.8) | 0.041 |
|  | p^c^ | - | 0.42 | 0.455 |  |
| constr%(SD), %over baseline | pre | -2.6(1.9) | -3.1(1.9) | -3.4(2.2) | 0.287 |
|  | post | - | -3.0(2.1) | -3.1(1.8) | 0.247 |
|  | p^c^ | - | 0.519 | 0.181 |  |
| tdila(SD), s | pre | 13.4(6.1) | 11.8(6.3) | 12.5(5.9) | 0.400 |
|  | post | - | 13.1(5.4) | 12.9(5.9) | 1.0 |
|  | p^c^ | - | 0.068 | 0.389 |  |
| tconstr(SD), s | pre | 50.3(25.5) | 50.5(28.2) | 50.9(23.7) | 0.959 |
|  | post | - | 50.3(27.5) | 48.0(26.8) | 0.303 |
|  | p^c^ | - | 0.766 | 0.327 |  |
| Vein |  |  |  |  |  |
| vessel number |  | 371 | 116 | 169 |  |
| Diameter(SD), MU | pre | 107.3(28.7) | 107.1(24.9) | 113.9(30.3) | 0.105 |
|  | post | - | 105.9(24.4) | 112.7(30.2) | 0.114 |
|  | p^c^ | - | 0.003 | 0.018 |  |
| dila%(SD), % over baseline | pre | 4.1(2.3) | 3.2(2.5) | 5.3(3.6) | <0.001 |
|  | post | - | 3.9(2.6) | 5.2(3.4) | <0.001 |
|  | p^c^ | - | 0.035 | 0.338 |  |
| constr%(SD), %over baseline | pre | -2.1(1.9) | -2.1(1.4) | -2.5(1.8) | 0.044 |
|  | post | - | -2.1(1.5) | -2.6(2.3) | 0.080 |
|  | p^c^ | - | 0.837 | 0.886 |  |
| tdila(SD), s | pre | 15.5(4.8) | 14.5(5.5) | 16.3(4.2) | 0.002 |
|  | post | - | 15.2(4.9) | 14.9(5.2) | 0.555 |
|  | p^c^ | - | 0.440 | 0.005 |  |
| tconstr(SD), s | pre | 49.3(35.9) | 55.1(35.5) | 57.9(32.9) | 0.846 |
|  | post | - | 50.4(35.4) | 53.3(34.0) | 0.462 |
|  | p^c^ | - | 0.186 | 0.231 |  |

^a^Quoted from our last publication. ^b^Mann-Whitney U-test or independent-samples T test for comparisons between reading and eye yoga groups. ^c^Wilcoxon test or paired T test for pre- and post-treatment comparison.
