# Supplementary material for: Eye yoga for glaucoma: recovery of vascular dysregulation and visual field function—a randomized controlled trial
Source: EPMA J. 2024 Dec 19;16(1):37–49. doi: 10.1007/s13167-024-00389-x (PMC11842685; doi:10.1007/s13167-024-00389-x)
Supplement: Supplementary file 2 — Supplementary file2 (DOCX 18 KB) [file 13167_2024_389_MOESM2_ESM.docx]

| **Supp. Table 2. Vessel parameters as a function of visual field defect depth** | | | | |
| --- | --- | --- | --- | --- |
|  |  | mild | moderate | severe |
| Artery |  |  |  |  |
| vessel number | | 257 | 29 | 12 |
| Pattern Deviation(SD), dB | pre | -1(2.3) | -7.7(1.4) | -14.5(3) |
|  | post | -1.4(4) | -4.3(4.6) | -11.7(5.7) |
|  | p ^†^ | 0.123 | <0.001 | 0.021 |
| Diameter(SD), MU | pre | 92.4(15) | 92.1(16.3) | 95.7(9.5) |
|  | post | 92(15.3) | 93(16.2) | 94.6(9.3) |
|  | p ^†^ | 0.041 | 0.244 | 0.265 |
| dila%(SD), % over baseline | pre | 3.8(2.8) | 4.2(3.2) | 4.6(3.1) |
|  | post | 3.6(2.9) | 3.7(2.9) | 5(4.5) |
|  | p ^†^ | 0.353 | 0.510 | 0.307 |
| constr%(SD), %over baseline | pre | -3.3(2.2) | -3.3(2.1) | -3(1.6) |
|  | post | -3(1.9) | -3.3(1.8) | -2.8(1.5) |
|  | p ^†^ | 0.163 | 0.837 | 0.802 |
| tdila(SD), s | pre | 42.2(6.2) | 42.7(5.4) | 42.4(5.4) |
|  | post | 43(5.5) | 40.6(6.7) | 43.9(5.8) |
|  | p ^†^ | 0.066 | 0.105 | 0.554 |
| tconstr(SD), s | pre | 80(25.5) | 86.3(26.1) | 83.9(29.4) |
|  | post | 79.4(26.8) | 73.1(25.9) | 68.5(37.3) |
|  | p ^†^ | 0.947 | 0.081 | 0.275 |
| Vein |  |  |  |  |
| vessel number | | 249 | 30 | 12 |
| Pattern Deviation(SD), dB | pre | -1(2.2) | -8.1(1.6) | -14.7(2.9) |
|  | post | -1.1(3.5) | -4(5.8) | -9.8(7.3) |
|  | p ^†^ | 0.405 | <0.001 | 0.008 |
| Diameter(SD), MU | pre | 111.2(28.8) | 109.5(29.3) | 115.6(21.8) |
|  | post | 110.2(28.3) | 109(30.4) | 113.8(22.4) |
|  | p ^†^ | 0.001 | 0.336 | 0.107 |
| dila%(SD), % over baseline | pre | 4.6(3.2) | 5(4.1) | 4.2(2.6) |
|  | post | 4.6(3.1) | 4.9(3.4) | 4.5(3.3) |
|  | p ^†^ | 0.807 | 0.766 | 0.65 |
| constr%(SD), %over baseline | pre | -2.3(1.7) | -2.6(1.8) | -1.9(1.3) |
|  | post | -2.3(1.9) | -2.8(2.6) | -2.6(1.6) |
|  | p ^†^ | 0.59 | 0.829 | 0.133 |
| tdila(SD), s | pre | 45.7(4.9) | 45.5(4.1) | 44.9(4.7) |
|  | post | 45.2(5.1) | 45.4(4.6) | 41.4(6.2) |
|  | p ^†^ | 0.162 | 0.698 | 0.039 |
| tconstr(SD), s | pre | 87.3(33.6) | 78.8(35.8) | 98.6(34.8) |
|  | post | 82.5(34.8) | 82.5(35.4) | 89.5(33.4) |
|  | p ^†^ | 0.075 | 0.489 | 0.48 |

†Wilcoxon test or paired T test for pre- and post-treatment comparison. Mild defect > − 6 dB, − 6–12 dB moderate defect and severe defect ≤ − 12 dB.
